# Visible-NIR hyperspectral classification of grass based on multivariate smooth mapping and extreme active learning approach

**DOI:** 10.1038/s41598-022-13136-x

**Published:** 2022-05-30

**Authors:** Xuanhe Zhao, Xin Pan, Weihong Yan, Shengwei Zhang

**Affiliations:** 1 College of Computer and Information Engineering, Inner Mongolia Agricultural University, Hohhot, 010018 China; 2 Institute of Grassland Research of CAAS, Hohhot, 010010 China; 3 College of Water Conservancy and Civil Engineering, Inner Mongolia Agricultural University, Hohhot, 010018 China; 4 Inner Mongolia Autonomous Region Key Laboratory of Big Data Research and Application of Agriculture and Animal Husbandry, Hohhot, 010018 China; 5 Key Laboratory of Water Resources Protection and Utilization of Inner Mongolia Autonomous Region, Hohhot, 010018 China

**Keywords:** Grassland ecology, Computer science

## Abstract

Grass community classification is the basis for the development of animal husbandry and dynamic monitoring of environment, which has become a critical problem to further strengthen the intelligent management of grassland. Compared with grass survey based on satellite remote sensing, the visible near infrared (NIR) hyperspectral not only monitor dynamically in a short distance, but also have high dimensions and detailed spectral information in each pixel. However, the hyperspectral labeled sample for classification is expensive and manual selection is more subjective. In order to solve above limitations, we proposed a visible-NIR hyperspectral classification model for grass based on multivariate smooth mapping and extreme active learning (MSM–EAL). Firstly, MSM is used to preprocess and reconstruct the spectrum. Secondly, by jointing XGBoost and active learning (AL), the advanced samples with the largest amount of information are actively selected to improve the performance of target classification. Innovation lies in: (1) MSM global enhanced preprocessing spectral reconstruction algorithm is proposed, in which isometric feature mapping is effectively applied to the grass hyperspectral for the first time. (2) EAL framework is constructed to solve the issue of high cost and small number for hyperspectral labeled samples, at the same time, enhance the physical essence behind spectral classification more intuitively. A field hyperspectral collection platform is assembled to establish nm resolution visible-NIR hyperspectral dataset of grass, Grass1, containing 750 samples, which to verify the effectiveness of the model. Experiments on the Grass1 dataset confirmed that compared with the full spectrum, the time consumption of MSM was reduced by 9.471 s with guaranteed overall accuracy (OA). Comparing EAL with AL, and other classification algorithms, EAL improves OA 22.2% over AL, and XAL has the best performance value on Kappa, Macro, Recall and F1-score, respectively. Altogether, the lightweight MSM–EAL model realizes intelligent and real-time classification, providing a new method for obtaining high-precision inter group classification of grass.

## Introduction

In China, abundant grassland resources, accounts for about 41 percent of the total land area^1^. Grassland plays a significant role in protecting the ecological environment, developing animal husbandry, and spreading grassland culture^2^. Ecosystems are damaged due to overgrazing, industrial manufacturing and natural disasters, causing environmental problems. In recent years, although the state has strengthened the management and maintenance of grassland resources, problems such as lacking of fine forage resources, grassland degradation, and conflicts between grass and livestock still endanger the balance of the ecosystem^3,4^. The classification of grass community is the basis for related researches such as dynamic monitoring of environmental changes and biomass estimation. It has become a core issue to further strengthen the intelligent management of grassland, and has far-reaching significance for realizing the sustainable development of grass resources.

Recently, the survey and monitoring of grassland is mainly based on satellite remote sensing, but it has certain limitations of low overall resolution and high cost. The high-resolution visible-NIR hyperspectral acquired at a close range can overcome above shortcomings. After the twenty-first century, with the continuous development and maturity of hyperspectral images (HSI) technology and related theories, it has broad application prospects in the field of grassland ecology^5^. Hyperspectral for parameter detection has the advantages of multiple bands, high sensitivity and non-destructiveness^6,7^. It facilitates grass classification with study at close range. McCann C. et al. applied HSI for quantitative comparison of variations in vegetation health and land. Classification using histograms of biophysical parameters to determine the main categories are presented in the dataset^8^. Marcinkowska-Ochtyra A. et al. have explored the different grass growth stages of Molinia caerulea and Calamagrostis epigejos with spectral bands and high spatial resolution. Using random forest (RF) classification, it was estimated that the best analysis dates of two species of grass were M. caerulea Kappa (0.85) in August and C. epigejos Kappa (0.65) in September^9^. Recently, Kang X. et al. adopted unmanned aerial vehicle HSI to predict the aboveground biomass of grassland, and quantifying the spectrum through characteristic parameters to ensure the prediction accuracy^10^. At present, grassland surveys mainly concentrate on coverage calculation and degradation, and there are few reports on hyperspectral identification of multiple types of grass.

The application of hyperspectral and machine learning has promoted the research and development of various intelligent recognition models^11^. Ai W. et al. applied HSI technology in the rapid identification of microplastics in farmland soil. The study established three models including decision tree (DT), support vector machine (SVM), and convolutional neural network (CNN). These results show that the CNN model based on the S-G smoothing filter obtains the best effect, the classification accuracy reached 92.6%^12^. Zhao X. et al. proposed a multi-step approach based on HSI and continuous wavelet analysis (CWA) to discriminate the plant stresses. The research constructed the identification model of the three tea plant stresses via the RF algorithm. The overall accuracy of the approach reached 90.26–90.69%^13^. Cui Y. et al. screened of maize haploid kernels based on near infrared spectroscopy quantitative analysis. The modeling is realized through partial least square (PLS) regression, and the average accuracy above 90%^14^. It can be seen that exploring an accurate and efficient classification model is still the focus of research.

Therefore, this study aims to classify the multi-category grasses in the field efficiently based on visible-NIR hyperspectral imaging technology and machine learning. We constructed the multivariate smooth mapping and extreme active learning (MSM–EAL) model, and achieved high-precision classification of grass species by optimizing it. Three parts are containing in the proposed model. Firstly, we assembled a hyperspectral field system to collect nm-level resolution HSI at the close-range to build a typical dataset of grass in the field. Then, we proposed a spectral reconstruction MSM algorithm to select representative spectral. Finally, the MSM–EAL model is established to achieve the timely and effective classification of grass. The novelty and contributions of this paper are as follows:

1. The field hyperspectral acquisition system was assembled to build a multi-category grass population near-ground HSI dataset Grass1.
2. A global enhanced preprocessing spectral reconstruction algorithm MSM was proposed to address the classic problems of feature selection and computational complexity of hyperspectral data. We reconstructed a relatively complete grass visible-NIR spectral dataset based on the smooth manifold projection technique Isomap. Furthermore, the result of full spectrum (FS) and MSM on the model were compared, validating the positive effect of MSM.
3. The EAL framework based active learning was constructed to solve the problem of small number and high cost for hyperspectral labeled samples, and alleviate the difficulty of model classification to a certain extent. Furthermore, it enhances the physical essence within spectral classification more intuitively. The self-constructed Grass1 dataset collected by our laboratory verified the validity of the MSM–EAL.

## Materials and methods

### Study area

Grassland herbage samples are from Shaerqin base, institute of grassland research of CAAS (Chinese Academy of Agricultural Sciences). We obtained the permission of the institution to take HSI of the grassland sample. Our work did not cause damage to grassland. Researcher Weihong Yan of the institute provided us with relevant information about grassland. The land use type in the study area is mainly grassland, which is composed of forage species, most of which are representative species of typical grassland. We take this area as an example to conduct research on grass classification. By enriching the relevant recognition technology, it can also be used as a reference for the pastures of other grasslands. The grass species Grass1 for the experiment is shown in Table 1. The official introduction of plant materials is detailed in the *flora of China*^15^.

**Table 1:** Samples information for Grass1 dataset.

NO	Name	Samples
C1	*Medicago sativa* L.cv.Aohan	50
C2	*Medicago ruthenica* Sojak cv. Zhilixing	50
C3	*Elymus canadensis* L.	50
C4	*Hordeum brevisubulatum* (Trin.) Link	50
C5	*Medicago varia* Martin. cv. Caoyuan No.3	50
C6	*Onobrychis viciaefolia* Scop. cv. Mengnong	50
C7	*Trifolium repens* L.	50
C8	*Melilotoides ruthenica*	50
C9	*Agropyron cristatum* (L.) Gaertn	50
C10	*Lespedeza bicolor* Turcz	50
C11	*Medicago falcata* L.	50
C12	*Elymus sibiricus* Linn	50
C13	*Avena sativa* L.	50
C14	*Festuca rubra* L.	50
C15	*Bromus ciliatus* L.	50
Total	–	750

### The field hyperspectral platform

We assemble a system for collecting HSI in the field: HyperSpec©PTU-D48E HSI instrument, high-precision scanning PTZ, tripod, data analysis software Hyperspec, etc. The light source is natural light. The imaging instrument is in line scanning mode. Table 2 shows the technical parameters.

**Table 2:** Technical parameters of hyperspectral instrument.

Index	Parameter
Spectrometer detector model	Andor Luca
PTZ/scanner serial port number	COM4
PTZ/scanner type	DP PTU-D48E
Spectral range/nm	400–1000
Number of spectral channels	750
Pixel mixing times	6
Band number	125
Spectral resolution/nm	4.8
Average times	3
Time of exposure/ms	12
Horizontal angle (°)	2.4
Tilt angle (°)	− 8.4
Starting angle (°)	− 15
Scan length (°)	30
Scanning step (°)	0.02
Number of scans	1499

### Data collection

**Figure 1:**
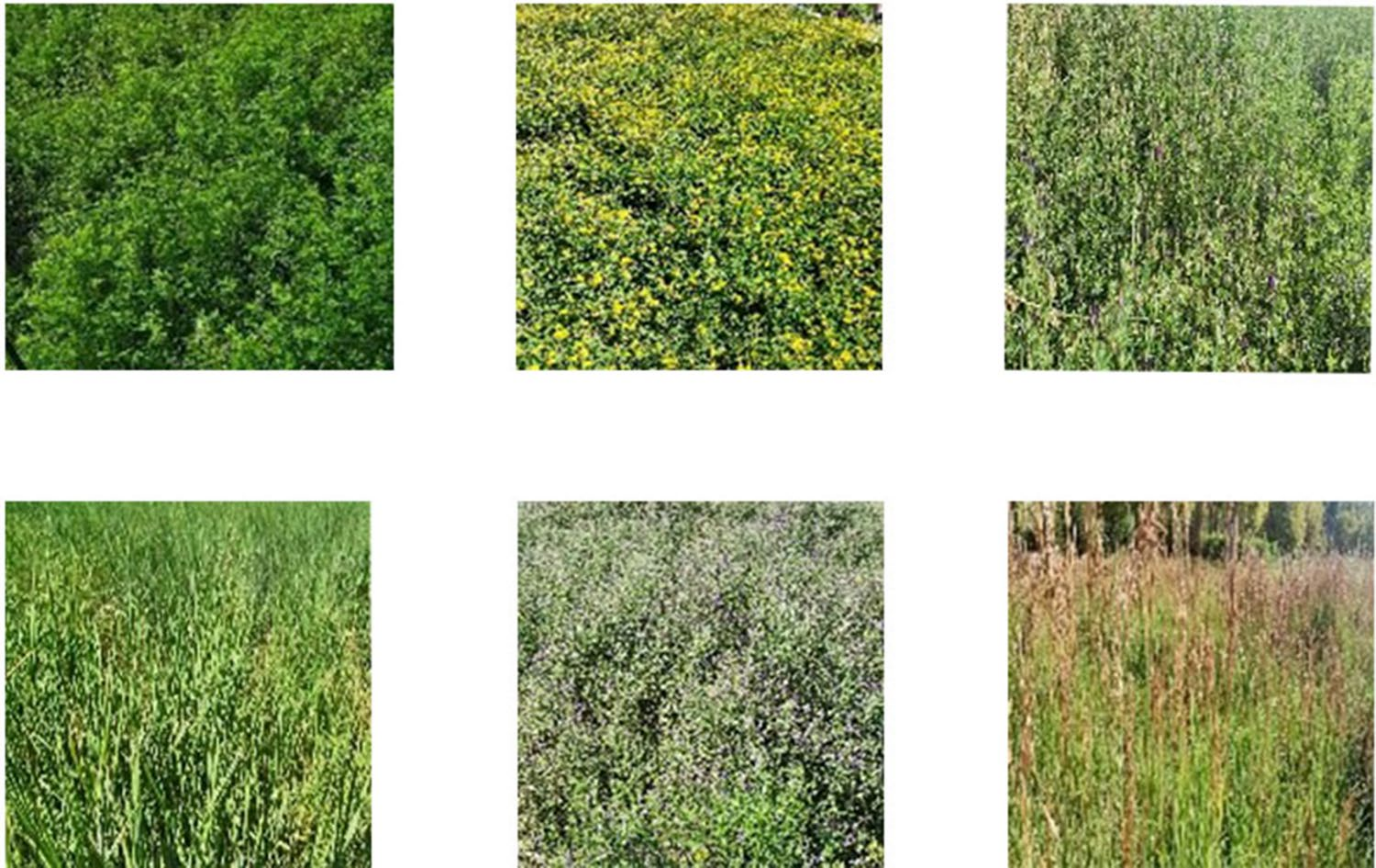
True color map of grass samples.

In July 2021, the data was collected during the lush grass growth period. Collect data from 11:00 a.m. to 2:00 p.m. every day. At this time, it is sunny, cloudless and the wind force does not exceed level 2. So as to ensure the consistency of the acquisition time line and avoid the influence of different degrees of light on the reflectivity as far as possible. The measuring points are arranged facing the sun and the opposite direction of the shadow. We collect data from different angles of the grassland, which is based on the growth of various types of forages, and selects relatively concentrated places within the study area. Each shot is a single category of grass. The image resolution is 1166 × 1004 pixels (Fig. 1). The imaging spectrometer is fixed with scanning head when shooting. Data acquisition and transmission are executed on Hyperspec software. Then save it as a BIL file. The ENVI5.3 software was used to extract the forage spectrum to establish the dataset Grass1. Well balanced regions with a clear image, uniform spectral distribution are selected for further segmentation. The average value of spectral reflectance of grass pixels was taken as the reflectance spectrum of a single type of grass.

### Methodology

**Figure 2:**
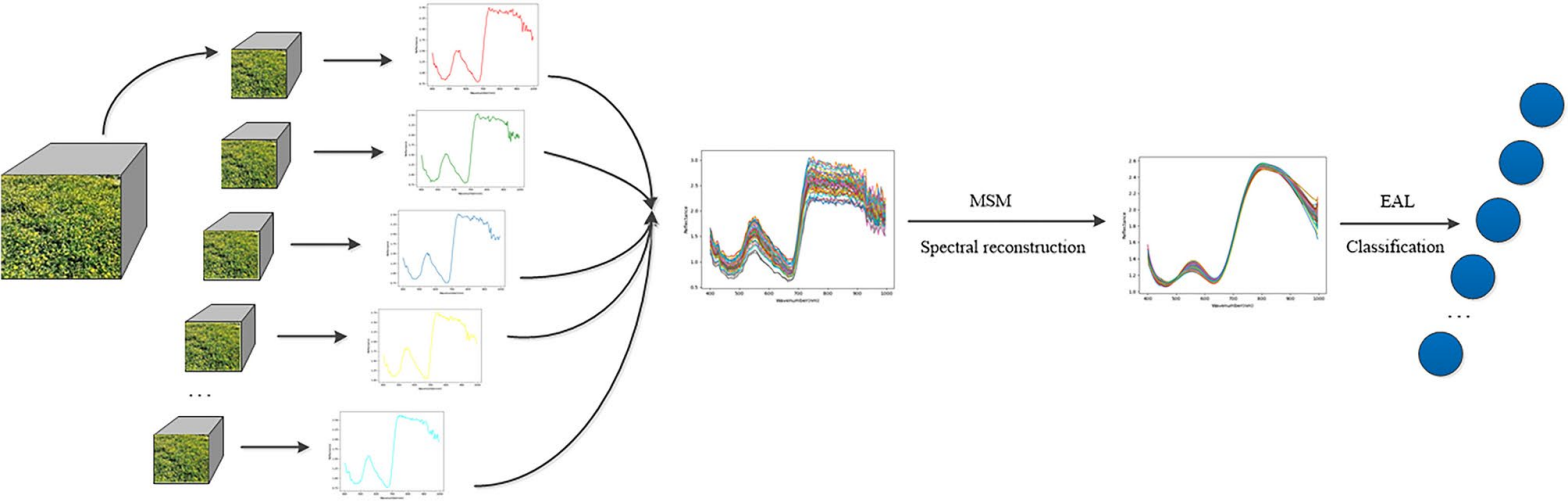
Proposed MSM–EAL framework for grass HSI classification.

In Fig. 2, we present the framework of visible-NIR hyperspectral classification of grass based on multivariate smooth mapping and extreme active learning (MSM–EAL). Specifically, we first introduce the proposed MSM algorithm for global enhanced spectral reconstruction, which utilizes smooth manifold projection technology to alleviate the problems of difficult feature selection and redundant data. Then, the EAL framework is proposed to address the matter of hyperspectral labeled samples and spectral classification. In the following, each step of this method will be presented in detail.

### The proposed MSM algorithm

In the process of field HSI acquisition, on the one hand, the surface distribution of grass is uneven and the plant height is different, causing certain scattering effect and coverage spectrum change. On the other hand, HSI is easy to be disturbed by external natural factors such as light, wind and shadow, resulting in a certain degree of distortion. Multiplicative scatter correction (MSC) is a scattering correction effect, which helps to eliminate the scattering effect caused by the above reasons and enhance the spectral variability. The moving window smooth spectral matrix (Nirmaf) belongs to the smooth effect, which improve the signal-to-noise ratio of the spectrum and reduce the influence of random noise^16,17^. Preprocessing methods are different and related to each other. We design an enhanced preprocessing multivariate smooth (MS) method that fusing MSC and smooth Nirmaf to target grass spectral signal features. In the follow-up, a model will be established to verify the validity of MS.

Most of the high-dimensional spatial data have the characteristics of being embedded in a manifold body, so the manifold learning isometric feature mapping (Isomap) based on spectral theory is adopted. Isomap preserves the global geometric features of the initial data and extracts features by reconstructing the underlying smooth manifold of HSI. It is nonlinear dimensionality reduction based on linear and multidimensional scaling transformation^18^. Isomap has been applied in image and HSI classification^19,20^, but there is no report on visible-NIR hyperspectral classification of grass.

In view of the above, we proposed the multivariate smooth mapping (MSM) spectral reconstruction algorithm, which can be represented as follows:

1 $$ MSM_{z} { } = { }\frac{{\left( {P_{j} - b_{j} } \right)\left( {2n + 1} \right) + n_{j} \cdot \mathop \sum \nolimits_{j = - n}^{n} C_{j} P_{k + j} }}{{n_{j} \left( {2n + 1} \right)}} + V_{Z} F_{Z}^{\frac{1}{2}} { } $$

where *P*_*j*_, *b*_*j*_, and *C*_*j*_ represent the raw reflectance value of spectrum *j*, baseline shift amount, and weight factor, respectively, *k* and *n*_*j*_ represent the polynomial degree and offset, respectively. *MSM*_*z*_ is the feature cube reconstructed to *Z* dimension from the spectrum calculated by 2*n + 1* moving window width, *V* eigenvector matrix and *F* eigenvalue matrix.

In Isomap equidistant mapping, the shortest path of edge *P*_*i*_* P*_*j*_ needs to be solved, and the representation matrix is:

2 $$ D_{G} = [d_{G}^{2} (P_{i} ,P_{j} )]_{i,j = 1}^{n} $$

where *d (P*_*i*_*, P*_*j*_*)* is the weight of the edge *P*_*i*_* P*_*j*_ calculated from the neighborhood graph *G* and its side *P*_*i*_* P*_*j*_.

### The proposed EAL framework

Labeling hyperspectral samples is expensive in terms of time and cost, at the same time, the lower spatial resolution and more bands increase the difficulty of labeling. Active learning (AL) provides an efficient labeling strategy, which only needs to label a relatively small number of samples to learn a more accurate model^21^. The pool-based AL selects the most informative samples according to the query strategy for limited labeling through iteration, so as to facilitate model improvement. Commonly used query strategies are uncertainty criteria, such as least confidence^22^, the bayesian active learning disagreement (BALD), the entropy sampling^23^, etc.

Due to there is still an over-fitting problem, different strategies such as hybrid prediction and regularization need to be used for non-recursive datasets^24^. The research^25^ proposed that extreme gradient boosting algorithm (XGBoost) based on gradient boosting. As a classification method, XGBoost has been successfully applied in Kaggle competition and other fields. Its most important feature for visible-NIR hyperspectral classification is that can easily and directly classify according to features, and the physical interpretation of features can help understand the electronic nature behind spectral classification. XGBoost is a machine learning algorithm based tree structure that integrates multiple weak classifiers to achieve flexible and high-precision classification. It is an upgraded version of gradient boosting decision tree. The optimization process of XGBoost entailed: (1) Expanding the objective function to the second order, and finds a new objective function for the new base model to improve the calculation accuracy. (2) L_2_ regularization term is added to the loss function to prevent over-fitting. (3) Using blocks storage structure realize automatic parallel computing^26,27^. The algorithm steps are as follows:

The objective function:

3 $$ L\left( \Phi \right) = \mathop \sum \limits_{i} l\left( {y^{i} ,\widehat{{y^{i} }}} \right) + \mathop \sum \limits_{k} \Omega \left( {f_{k} } \right) $$

In formula (3), the first and second terms are the loss function term and the regularization term, respectively. Where,

4 $$ \Omega \left( {f_{k} } \right) =\upgamma {\text{T}} + \frac{1}{2}\lambda \left\| w \right\|^{2} $$

*γ* and *λ* are regularization parameters which are used to adjust complexity of the tree.

Next, second derivative Taylor expansion of the objective function. Where $g_{i}$ and $h_{i}$ are the first derivative and second derivative, respectively.

5 $$ L^{\left( t \right)} = \mathop \sum \limits_{i = 1}^{n} l\left( {y_{i} ,\widehat{{y_{i}^{t - 1} }} + f_{t} \left( {x_{i} } \right)} \right) + \Omega \left( {f_{t} } \right) $$

6 $$ g_{i} = \partial_{{\hat{y}_{i} (t - 1)}} l\left( {y_{i} ,\widehat{{y_{i}^{t - 1} }}} \right) $$

7 $$ h_{i} = \partial_{{\widehat{{y_{i} }}(t - 1)}}^{2} l\left( {y_{i} ,\widehat{{y_{i}^{t - 1} }}} \right) $$

8 $$ {\text{L}}^{\left( t \right)} \approx \mathop \sum \limits_{i = 1}^{n} \left[ {l\left( {y_{i} ,\widehat{{y_{i}^{t - 1} }}} \right) + g_{i} f_{i} \left( {x_{i} } \right) + \frac{1}{2}h_{i} f_{t}^{2} \left( {x_{i} } \right)} \right] + \Omega \left( {f_{t} } \right) $$

Final objective function:

9 $$ {\hat{\text{L}}}^{ i} \left( q \right) = - \frac{1}{2}\mathop \sum \limits_{j = 1}^{T} \frac{{(\mathop \sum \nolimits_{{i \in I_{j} }} g_{i} )^{2} }}{{\mathop \sum \nolimits_{{i \in I_{j} }} h_{i} + \lambda }} + \gamma T $$

Equation (9) can be used as the fraction of tree cotyledons, and the tree structure is directly proportional to the fraction. If the result after splitting is less than the maximum value of the given parameter, the cotyledon depth stops growing^24,28^.

AL solves the problems of limited number and high cost of grass hyperspectral labeling samples. The default model of traditional AL is logistic regression, which is mostly studied on the ideal public dataset. However, the actual data has more uncertain noise, which still poses a certain challenge to AL. Consequently, we propose the extreme active learning (EAL) framework to minimize the classification cost of visible-NIR hyperspectral. The framework replaces the logistic regression model with XGBoost. Taking advantage of AL, XGBoost can improve performance with less training marker samples. By jointing of XGBoost and AL, EAL provides significantly better results than AL in field Grassl dataset recognition. Additionally, based on the characteristics of XGBoost, EAL more intuitively enhances the physical essence behind spectral classification than AL. Algorithm 1 summarizes the workflow of EAL framework.
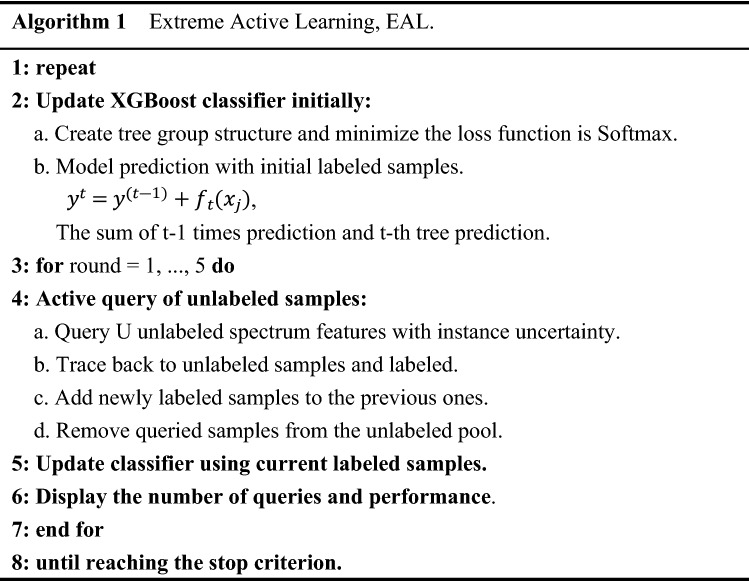


Random forest (RF) and decision tree (DT) were used to compare with EAL. RF and DT are frequently used in the field of grassland remote sensing^9,29^. Furthermore, RF, DT and XGBoost have the same point is that are learning algorithms based on tree structure. DT determines the direction by judging the conditions of the decision node^12^. RF is an integrated learning of multiple decision trees^30^.

## Experimental results

All experiments use PyCharm2019.2.5, python (3.8.8) performed on Intel(R) Core(TM) i5-6500, 3.20 GHz CPU, 8 GB RAM, which is provided by the Center of Information and Network Technology of Inner Mongolia Agricultural University. The established Grass1 visible-NIR hyperspectral dataset is used to evaluate the performance of MSM–EAL model. All quantitative comparisons used three commonly evaluation indicators, namely overall accuracy (OA), kappa coefficient and time-consuming. The results reported are the average of 5 runs. In each run, the initial labeled samples are randomly without fixing the random seeds. The statistical tests of confusion matrix (CM), Recall rate, Macro and F1-score were also carried out.

### MSM reconstruction spectrum

**Figure 3:**
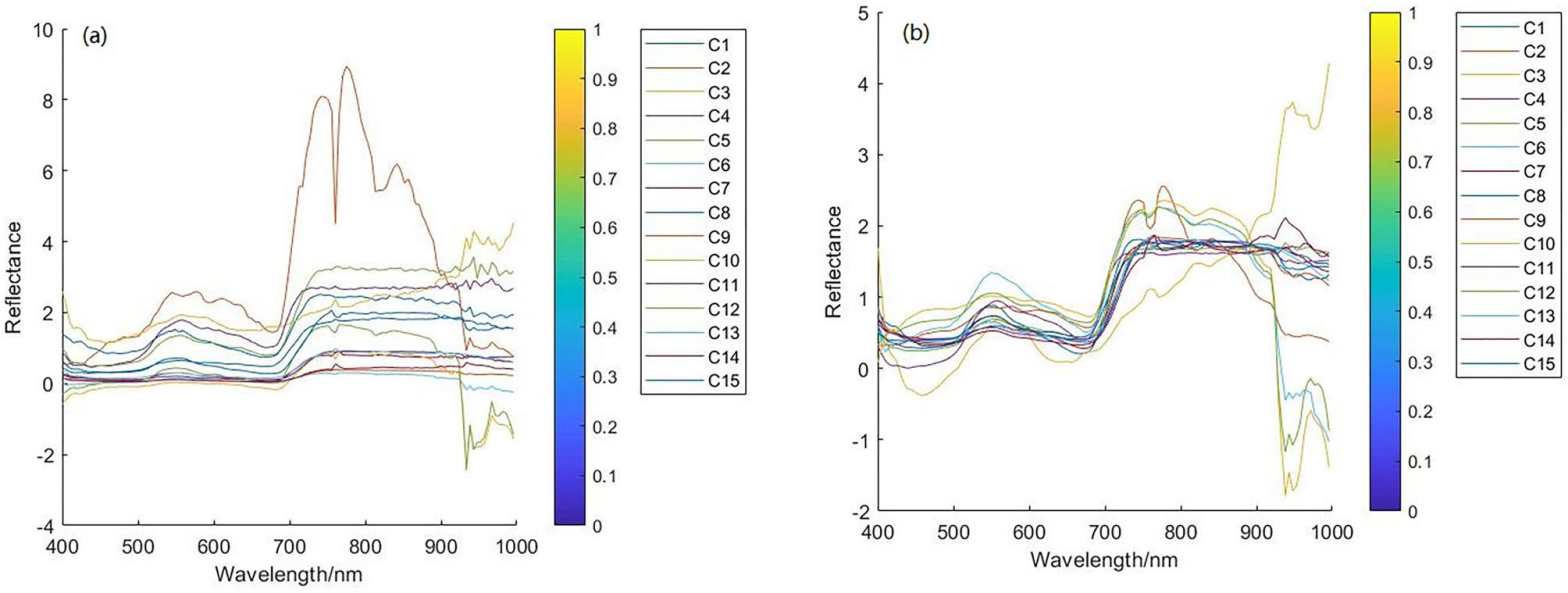
The average reflectance spectral curve of Grass1 (**a** raw, **b** MS).

MSM implements the MS optimization spectrum for the various grasses original spectrum of 400–1000 nm (Fig. 3). From horizontal analysis, each spectrum is interleaved. At 400–900 nm, the variation trend of the original spectral curve of 15 species of grasses is unanimous. Among them, the spectra overlap seriously at 440–690 nm, and there are similarities among C7, C10 and C13 spectra at 691–890 nm. At 900–1000 nm, there are two trends in the original spectral curve. The first (C2, C6, C10, C12) spectrum decreases. The second (C1, C3-C5, C7-C9, C11, C13-C15) spectrum increased. From longitudinal analysis, the spectral reflectance of C2 is the highest, which is about 933 nm. C2, C6, C10 and C12 produce troughs at the same position. C1, C3–C5, C7–C9, C11 and C13–C15 produce peaks at about 940 nm. It can be seen that the spectra of different types of grasses are different, but the positions of peak or trough are the same. After MS processing, the spectral shape changes to a certain extent, which reduces the error caused by spectral drift and increases the correlation and smoothness between data. MS makes the absorption peak of the spectrum more obvious and maintains great similarity with the original spectrum shape, which lays a foundation for the realization of spectral quantitative analysis.

**Figure 4:**
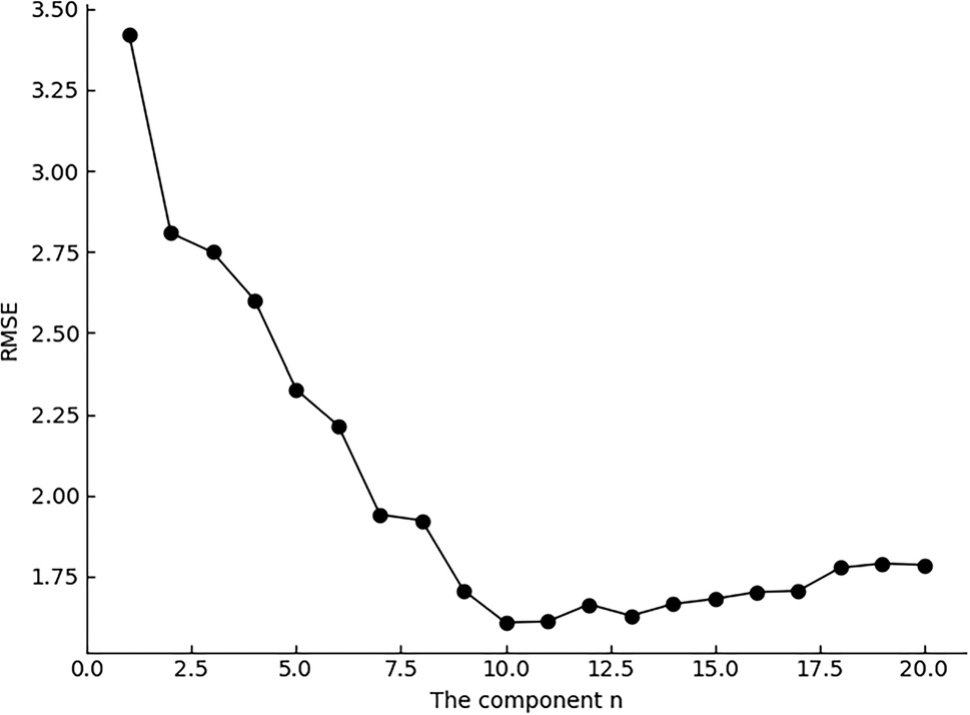
The RMSE with different components.

The essential of MSM spectrum reconstruction is the value of dimension. The setting range of dimension components is 1–20, and the optimum is determined according to the root mean square error (RMSE). In Fig. 4, the RMSE with smallest value 1.608 lies in 10 components. Simultaneously, the reconstructed spectrum is highly similar to the original one.

### MSM–EAL classification

MSM–EAL model mainly includes the following two parts. AL is used to implement the sample labeling strategy. Relevant important parameters are set as follows. The samples selection criteria is the query instance uncertainty, which selects the sample with the least confidence in the predicted value as the query instance. The smaller confidence of data is more difficult to distinguish, so it has more labeling value. The number of iterations is 5. When the number of queries equal 60, it is set as the stop criterion. The initialize label pool set 9.90%, i.e. 52. Each class contains at least one instance. Another 473 samples were randomly selected and set as unlabeled sample pool. The remaining 30% of dataset was reserved for testing.

XGBoost is used for classification. Use XGB classifier and automatically optimize parameters through Gridsearch. Adjust and optimize all important parameters before experimental settings (Table 3).

**Table 3:** The optimal parameters of XGBoost.

Parameter	Setting
Booster	gbtree
N estimators	160
Max depth	5
Min child weight	1
Subsample	0.6
Colsample bytree	0.6
Reg alpha	1e−05
Reg lambda	1
Eta	0.1
Learning rate	0.1
Nthread	4
Scale pos weight	1
Seed	27
Num. class	15

MSM–EAL model was established and compared with MSC, Nirmaf and FS to verify its effectiveness (Table 4). The evaluation indicators are OA, kappa and time consuming. The results shown that, (1) Contrast with MSC and Nirmaf, OA of MS increased by 16% and 17.3%, respectively, indicating the scientific rationality of MS grass spectral pretreatment method. (2) Comparing FS and MSM, the former has many bands and large memory consumption. If it is classified directly, it will increase the time complexity. The latter obtains representative and comprehensive features after spectral reconstruction. MSM operation speed is improved that time consumption reduced 9.471 s under the condition of ensuring accuracy. And MSM–EAL has the highest OA of 96.8%. The results confirm that MSM fits for spectral processing.

**Table 4:** EAL framework classification results after different spectral processing.

Method	OA/%	Kappa	Time/s
MSC-FS-EAL	80.8	0.794	60.822
Nirmaf-FS-EAL	79.5	0.780	303.146
MS-FS-EAL	96.8	0.966	57.660
MSC-Isomap-EAL	80.8	0.794	50.475
Nirmaf-Isomap-EAL	79.5	0.780	47.187
MSM–EAL	96.8	0.966	48.189

In this study, an active extreme gradient classification strategy EAL is proposed to solve the problems of hyperspectral data limited labeling and classification effect. Based on Table 5, the EAL framework has better classification ability than AL, which the OA increased by 22.2%, and has achieved good performance in five general indicators. Although the large number of EAL network parameters requires more time consuming, it can be accepted for the obviously improved accuracy. Subsequently, the comparative experiment was conducted with RF and DT. RF and DT have the same number of labeled samples as EAL and AL. Overall, EAL has certain advantages over AL, RF, and DT in classifying HSI with limited labeled samples under the same spectral dimension. In addition, it also verifies the importance of learning when the information of the sample is restricted.

**Table 5:** Comparison of classification results with the EAL, AL, RF and DT algorithms.

Method	OA/%	Kappa	Macro	Recall	F1	Time/s
EAL	96.8	0.966	0.966	0.969	0.968	48.189
AL	74.6	0.726	0.641	0.712	0.680	11.544
RF	52.0	0.489	0.453	0.569	0.439	3.284
DT	50.6	0.473	0.514	0.540	0.448	1.079

## Discussion

The parameters of the grass visible-NIR hyperspectral classification model based on multivariate smooth mapping and extreme active learning are carefully selected. The performance of the proposed method MSM–EAL, is tested from six aspects of OA, Kappa, Macro, Recall, F1 and Testing time. The experimental results on Grass1 dataset show the precision and stability of MSM–EAL, whose recognition effect is substantially better than some existing advanced algorithms^9,29^ (Tables 4, 5). This suggests that MSM–EAL is suitable for grass visible-NIR hyperspectral classification. The specific reasons are as follows.

**Figure 5:**
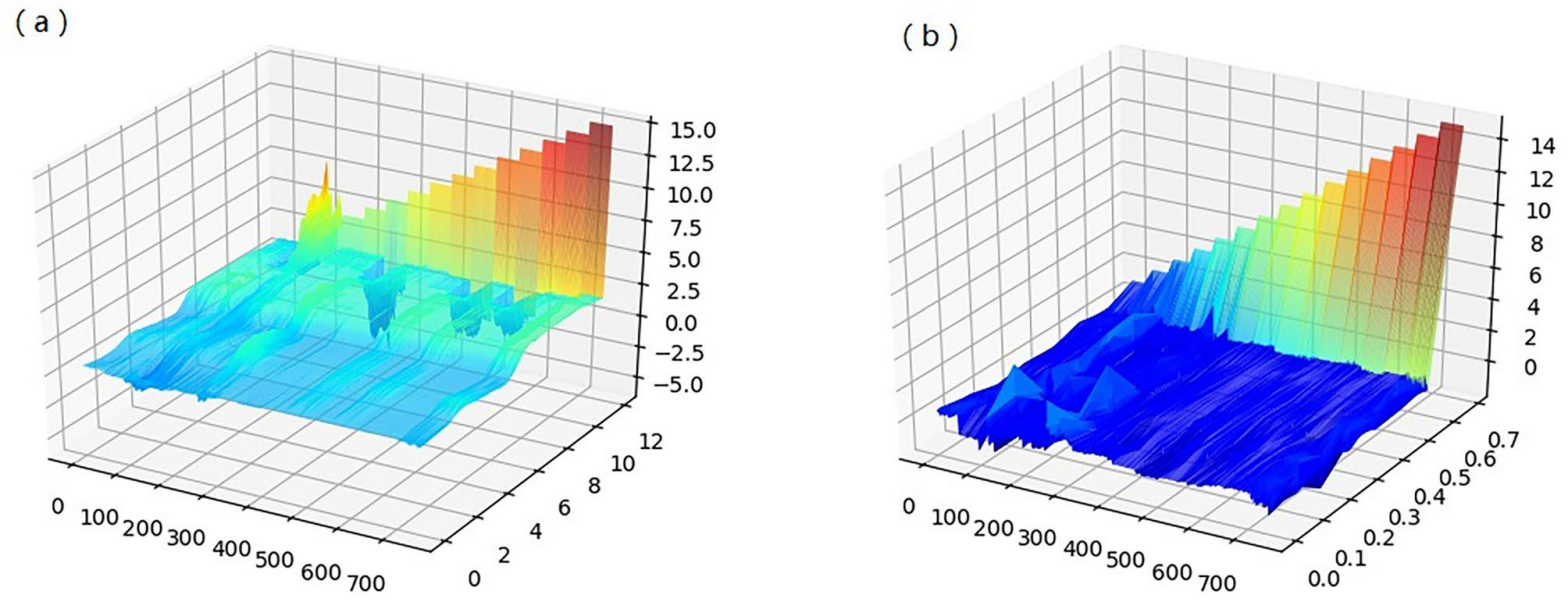
3D map of spectral feature (**a** raw spectrum, **b** MSM reconstructed spectrum).

According to the high-dimensional characteristics of hyperspectral data, a MSM spectral reconstruction algorithm is proposed. The structural features of low dimensional bottom manifold are extracted by Isomap to obtain the best spectral set and simplified model. The visualization effect before and after MSM spectral reconstruction is shown in Fig. 5. The data structure is reduced in the same proportion, the intra class distance is shortened, and the clustering effect and inter class separability are enhanced. The data distribution shows some linear laws with less overlap. The essential characteristics of grass have been better extracted after MSM, which alleviates the time complexity of high-dimensional data on the model.

**Figure 6:**
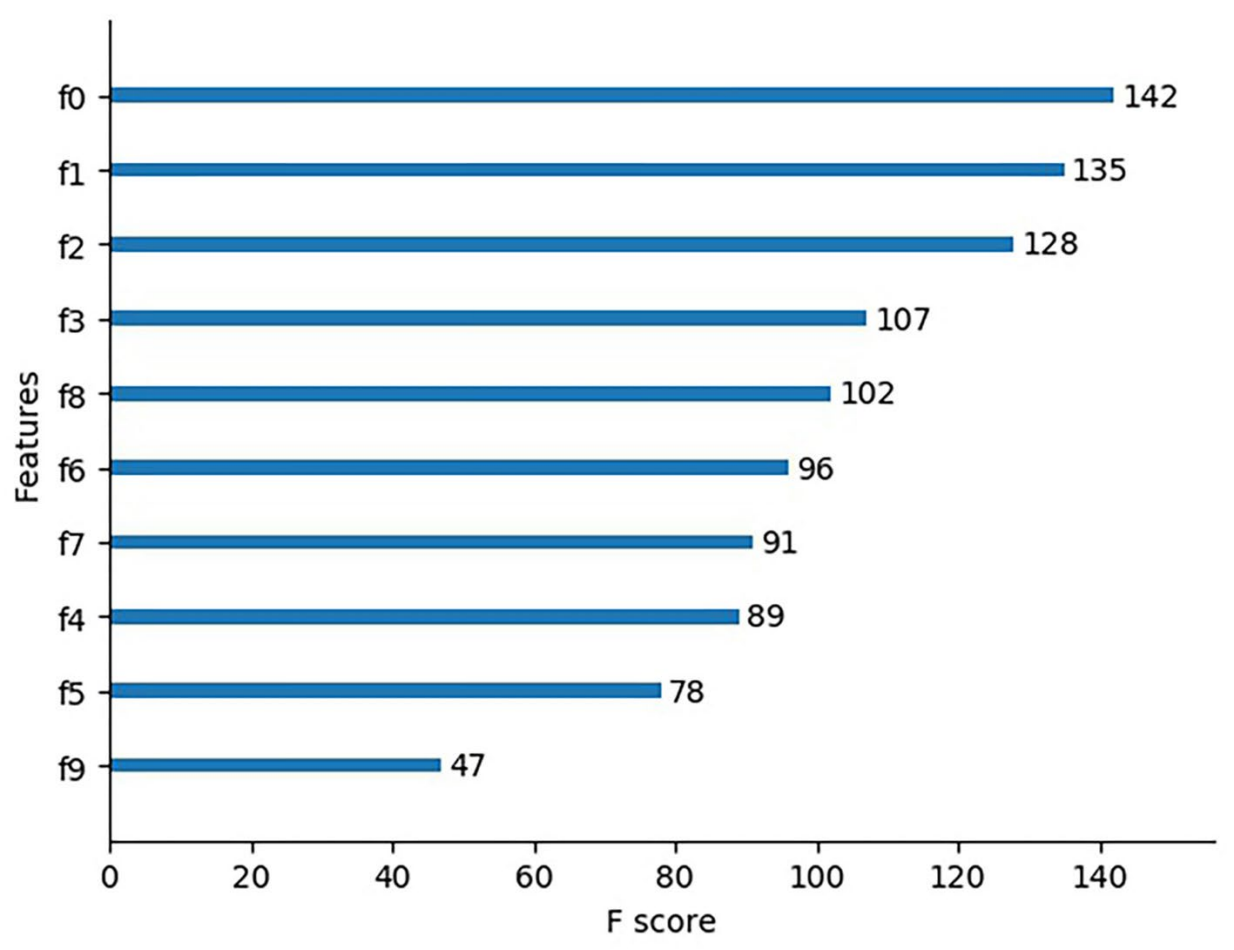
Ranking of feature importance scores after spectrum reconstruction.

XGBoost redefines the objective function by optimizing the loss function term with second order Taylor and adding L_2_ regularization term to prevent over fitting problem. Meanwhile, it helps to understand the physical essence of the features behind spectral classification. In MSM–EAL, all the 10 reconstructed features have high importance scores, of which f0 being the most important (Fig. 6). MSM reconstructs data of the manifold spectral features, removes the data in the sample set that does not contribute significantly to distinguishing samples, and obtains typical features. The above factors improve the accuracy of spectral classification.

**Figure 7:**
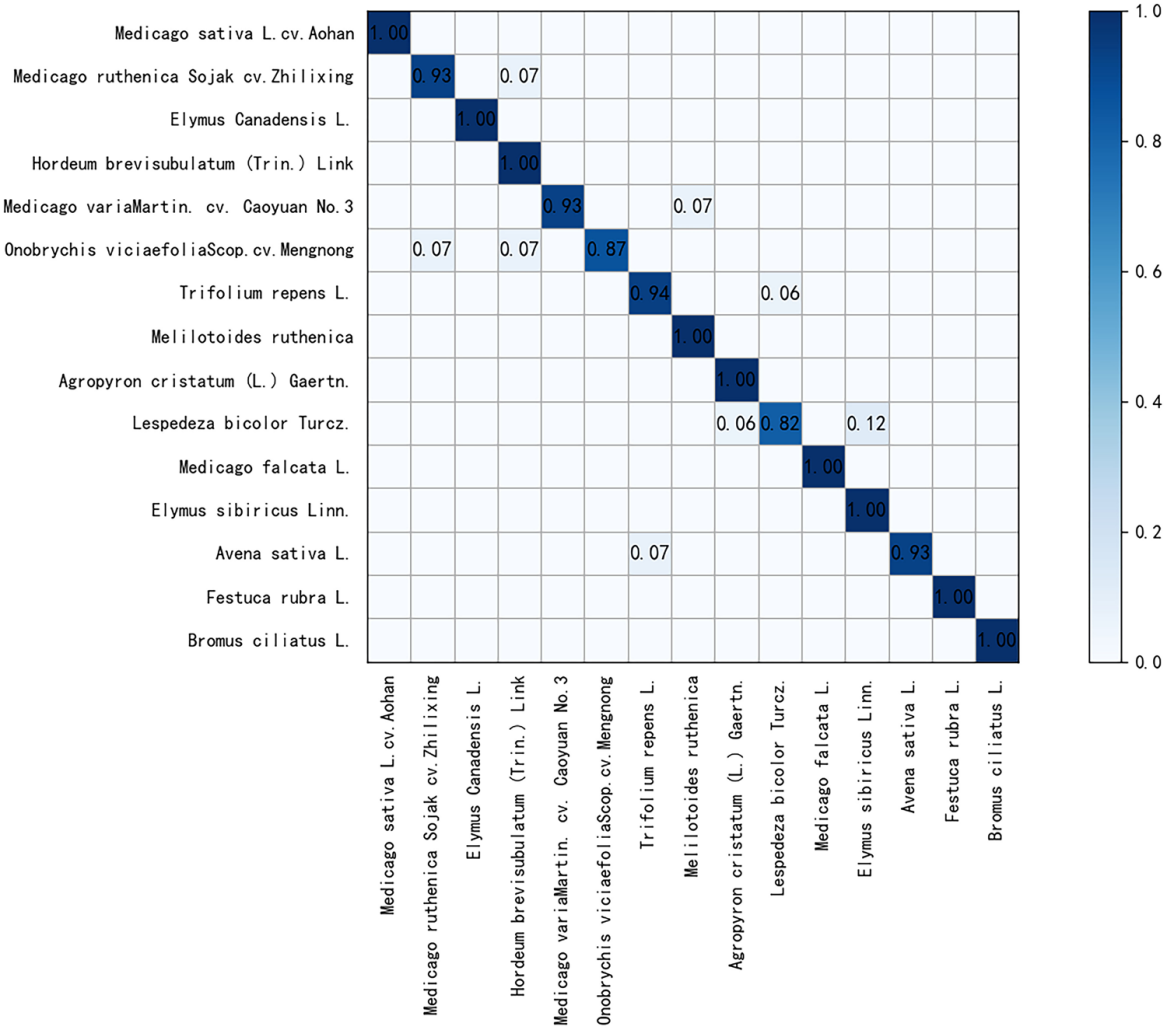
CM of the Grass1 dataset in MSM–EAL model.

Figure 7 shows the confusion matrix (CM) of 15 grass species in the Grass1 dataset. The classification accuracy of 87% category and 60% category grasses reached more than 90% and 100%, respectively. It indicates that the proposed model can better learn the spectral characteristics of various ground objects. The accuracy of Lespedeza bicolor Turcz is relatively low, 82%, because its internal structure spectrum is slightly similar to Elymus sibiricus Linn and Agropyron cristatum (L.) Gaertn., which is easy to be confused in recognition. However, the global average classification accuracy is more than 96%. Consequently, this model plays a positive role in the classification of highly similar grass categories.

## Conclusions

In this study, the MSM–EAL classification model was proposed and verified to enrich the hyperspectral research methods of multi category grasses and explore a micro intelligent visible-NIR hyperspectral classification model. MSM–EAL fully captures the essential spectral characteristics of grasses. The experimental evaluation of the established Grass1 dataset shows that the model has well recognition ability, the OA is 96.8%, which can be applied to the quantitative analysis of visible-NIR spectra of grasses. The novelty of this study is as follows: (1) a multi-category visible-NIR hyperspectral dataset Grass1 is established. (2) A global enhanced preprocessing spectral reconstruction algorithm MSM is proposed, which effectively extends the smooth manifold projection Isomap to the field of grass hyperspectral. (3) We construct EAL framework based on AL to solve the issue of limited labeled samples in grass hyperspectral classification. Simultaneously, more intuitively enhance the physical essence behind spectral classification.

So far, the classification of grass community by visible-NIR hyperspectral is still in infancy. In all quantitative comparisons, adding grass categories can improve the richness of datasets, but it has high requirements for classifiers. The balance between the two factors still needs to be discussed. Furthermore, MSM–EAL needs to be further optimized and the impact of training sample ratio on classification performance needs to be evaluated.

## Data Availability

The datasets generated and analyzed during the current study are not publicly available due that we have signed a confidentiality agreement with correlation department. At present, the project has not been completed as a whole. We have no right to public relevant hyperspectral data sets. However, it can be obtained from the corresponding author on reasonable request. Our study complies with Inner Mongolia Autonomous Region of China and China guidelines. It is supported by national, central and local funds.
